# Development and validation of a nomogram to predict the risk of sepsis-associated encephalopathy for septic patients in PICU: a multicenter retrospective cohort study

**DOI:** 10.1186/s40560-024-00721-7

**Published:** 2024-02-20

**Authors:** Guan Wang, Xinzhu Jiang, Yanan Fu, Yan Gao, Qin Jiang, Enyu Guo, Haoyang Huang, Xinjie Liu

**Affiliations:** 1 Department of Pediatrics, Qilu Hospital of Shandong University, No. 107 West Wenhua Road, Jinan, 250012 Shandong China; 2 Qilu Hospital of Shandong University, No. 107 West Wenhua Road, Jinan, 250012 Shandong China; 3 Department of Medical Engineering, Qilu Hospital of Shandong University, No. 107 West Wenhua Road, Jinan, 250012 Shandong China; 4 Department of Pediatrics, Jinan Children’s Hospital of Shandong University, No. 23976 Jingshi Road, Jinan, 250000 Shandong China; 5 Department of Pediatrics, Jining First People’s Hospital, No. 6 JianKang Road, Jining, 272000 Shandong China; 6 School of Public Health of Shandong University, No. 44 West Wenhua Road, Jinan, 250000 Shandong China

**Keywords:** Sepsis-associated encephalopathy, Nomogram, Prediction, PICU

## Abstract

**Background:**

Patients with sepsis-associated encephalopathy (SAE) have higher mortality rates and longer ICU stays. Predictors of SAE are yet to be identified. We aimed to establish an effective and simple-to-use nomogram for the individual prediction of SAE in patients with sepsis admitted to pediatric intensive care unit (PICU) in order to prevent early onset of SAE.

**Methods:**

In this retrospective multicenter study, we screened 790 patients with sepsis admitted to the PICU of three hospitals in Shandong, China. Least absolute shrinkage and selection operator regression was used for variable selection and regularization in the training cohort. The selected variables were used to construct a nomogram to predict the risk of SAE in patients with sepsis in the PICU. The nomogram performance was assessed using discrimination and calibration.

**Results:**

From January 2017 to May 2022, 613 patients with sepsis from three centers were eligible for inclusion in the final study. The training cohort consisted of 251 patients, and the two independent validation cohorts consisted of 193 and 169 patients. Overall, 237 (38.7%) patients developed SAE. The morbidity of SAE in patients with sepsis is associated with the respiratory rate, blood urea nitrogen, activated partial thromboplastin time, arterial partial pressure of carbon dioxide, and pediatric critical illness score. We generated a nomogram for the early identification of SAE in the training cohort (area under curve [AUC] 0.82, 95% confidence interval [CI] 0.76–0.88, sensitivity 65.6%, specificity 88.8%) and validation cohort (validation cohort 1: AUC 0.80, 95% CI 0.74–0.86, sensitivity 75.0%, specificity 74.3%; validation cohort 2: AUC 0.81, 95% CI 0.73–0.88, sensitivity 69.1%, specificity 83.3%). Calibration plots for the nomogram showed excellent agreement between SAE probabilities of the observed and predicted values. Decision curve analysis indicated that the nomogram conferred a high net clinical benefit.

**Conclusions:**

The novel nomogram and online calculator showed performance in predicting the morbidity of SAE in patients with sepsis admitted to the PICU, thereby potentially assisting clinicians in the early detection and intervention of SAE.

**Supplementary Information:**

The online version contains supplementary material available at 10.1186/s40560-024-00721-7.

## Introduction

Sepsis is a major cause of morbidity and mortality in children worldwide [1]. Sepsis-associated encephalopathy (SAE) is a diffuse brain dysfunction that excludes direct central nervous system infections, structural abnormalities, and metabolic encephalopathy [2–4]. Patients with SAE are characterized by a series of neurological disturbances, ranging from drowsiness and mild delusions to coma, and relatively rarely by convulsions, tremors, or myoclonus. Owing to the differences in the study populations, different studies have reported its incidence to be from 8% to more than 70% [5]. Patients with SAE tend to have a higher 28-day mortality, spend more time on a ventilator, and have a longer intensive care unit (ICU) stay [6]. Moreover, long-term cognitive impairment and functional disability can persist in many survivors of severe sepsis [7]. SAE can be an early feature of infection and appear before other systemic manifestations of sepsis [4]. As SAE is not a direct infection of central nervous system, the treatment focus remains on the proper management of sepsis [4]. Therefore, early identification of SAE, even before sepsis is diagnosed, is critical for timely investigation for and intervention of infection, thus reducing the associated morbidity and mortality.

Patients with SAE often have significantly higher heart rate, blood lactate, and serum sodium levels, as well as lower platelet count, serum albumin, and serum pH than patients without SAE [6, 8]. Some evidence suggests that electroencephalography (EEG) and magnetic resonance imaging (MRI) are useful for detecting the occurrence of SAE and assessing brain injury, but further clinical investigations are needed to confirm this conclusion [9, 10]. To date, most of the aforementioned research on SAE prediction has been performed in adults, whereas studies on pediatric patients are relatively lacking. Developing a prediction model that is widely used in critically ill children admitted to the pediatric intensive care unit (PICU) can contribute to a decline in the morbidity and mortality of pediatric patients with SAE.

The nomogram model, as a visualization tool, has been widely used in clinical prognosis research among critically ill and cancer patients [11]. In the present study, we collected multiple data points from patients with sepsis admitted to the PICU in three medical institutions to screen the risk factors of SAE and developed a novel prediction nomogram for the morbidity of SAE.

## Methods

### Study design and participants

We conducted a multicenter retrospective observational study involving the patients with sepsis admitted to the PICU of three hospitals in China from January 2017 to May 2022. Patient inclusion criteria were as follows: (1) age > 28 days and ≤ 18 years; (2) diagnosis of sepsis according to the sepsis 3.0 definition evaluated by pediatric sequential organ failure assessment scores (pSOFA) [12], which is the life-threatening organ dysfunction caused by a dysregulated host response to infection [13]. Patients with the following underlying conditions that may affect brain function and symptomatic diagnosis were excluded: (1) primary central nervous system disease, including traumatic brain injury, cerebral vascular disease, intracranial infection, autoimmune encephalitis, and epilepsy; and (2) metabolic encephalopathy caused by diabetic ketoacidosis, hypoglycemia, hepatic encephalopathy, and inherited metabolic diseases. Patients treated with mechanical ventilation during the first 24 h after PICU admission and patients with missing laboratory data were also excluded. This study was approved by the Institutional Ethics Review Board of Qilu Hospital of Shandong University (KYLL-202202-027-1). The requirement for individual patient consent was waived due to the retrospective design of the study, and all patient information was handled anonymously. The Transparent Reporting of a multivariable prediction model for Individual Prognosis or Diagnosis (TRIPOD) statement was used as reporting guideline (Additional file 1: Table S1) [14].

### Data collection

All data were reviewed and extracted from the electronic medical record systems of the three hospitals by a single data collector who was blinded to the study design. For each patient included in the study, the following variables were collected: (1) demographic data including age and gender; (2) the first data of vital signs during the first 2 h after PICU admission; (3) suspected infection site and micro-organism; (4) the use of vasopressors (adrenaline, norepinephrine, dopamine, dobutamine, and other vasopressors) during PICU stay time; (5) advanced life support during PICU stay time, including mechanical ventilation and renal replacement therapy; (6) outcomes, including the occurrence of multi-organ dysfunction and septic shock, hospital stay time, PICU stay time, and in-hospital mortality; (7) severity scores on PICU admission, namely the pediatric critical illness score (PCIS) [15]; (8) the first laboratory data after PICU admission. All patients underwent routine laboratory tests upon admission to PICU. For patients treated with renal replacement therapy during the first 24 h after PICU admission, we used the laboratory data before the treatment.

### Sepsis-associated encephalopathy

SAE is defined as the combination of sepsis with an altered mental status with cognitive or behavioral abnormalities accompanied by Glasgow Coma Scale (GCS) ≤ 14 during PICU stay [2, 4]. Cognitive and neuropsychiatric disorders of SAE include inattention, disorientation, agitation, irritability, decreased psychomotor activity, somnolence, stupor, and coma, which were clearly documented in medical record by doctors and nurses. Patients with changes in consciousness caused by primary central nervous system disease, metabolic encephalopathy, and toxicosis were excluded. For patients treated with medications that may cause a decrease in level of consciousness (e.g., sedative or analgesic drugs), clinical examination as well as neuroimaging, neurophysiological testing and serum biomarkers such as neuron-specific enolase (NSE) and S100 calcium-binding protein B (S100B) were used together to evaluate the diagnosis of SAE. Brain computer tomography (CT) and MRI findings vary from normal in mild cases of SAE to non-specific structural changes, such as vasogenic edema, leukoencephalopathy, ischemic lesions, and changes in subcortical and cerebellar regions in serious cases [16, 17]. EEG alterations in SAE patients include slow waves, triphasic waves, focal seizures, generalized suppression, and burst suppression [17]. Elevated serum levels of NSE and S100B are proven to be associated with brain injury in patients with sepsis [18], and the thresholds are 16.3 ng/mL for NSE and 0.105 µg/L for S100B in our centers. We checked the daily data in electronic records and once the diagnostic criteria were met, SAE could be diagnosed.

### Statistical analysis

Shapiro–Wilk tests were used to test the normal distributions of the variables. Continuous variables with normal distribution were tested by unpaired Student’s t test and presented as the mean ± standard deviation (SD), whereas non-normal distributed continuous variables were tested by Mann–Whitney U-test and presented as the median (interquartile range, IQR). Categorical variables were tested using Chi-square analysis or Fisher’s exact test and are described as numbers (percentages). Univariate and multivariate regression analyses were used to identify independent risk factors associated with SAE in the training cohort. Variables were omitted when more than 30% of the values were missing. Multicollinearity between continuous variables was detected by the variance inflation factor (VIF), and an arithmetic square root of VIF ≤ 2 was considered as non-collinearity.

For the model-building process, least absolute shrinkage and selection operator (LASSO) regression was used for variable selection and regularization in the training cohort. The selected variables were used to build a nomogram to predict the risk of SAE in patients with sepsis admitted to the PICU. The nomogram performance was assessed using discrimination and calibration. We used the receiver operating characteristic curve to assess the discriminative performance of the nomogram and then assessed the area under the curve (AUC). Calibration was performed using the bootstrap method with 1000 re-samplings to analyze the association between the observed incidence and predicted probability. We evaluated the clinical usefulness and net benefit of the new predictive model by decision curve analysis (DCA) in the training set and the validation sets. All statistical analyses were performed using R version 3.6.3 (https://www.r-project.org; R Foundation for Statistical Computing, Vienna, Austria). Statistical significance was defined as a two-sided *P*-value < 0.05.

## Results

### Baseline and clinical characteristics of the cohorts

**Fig. 1:**
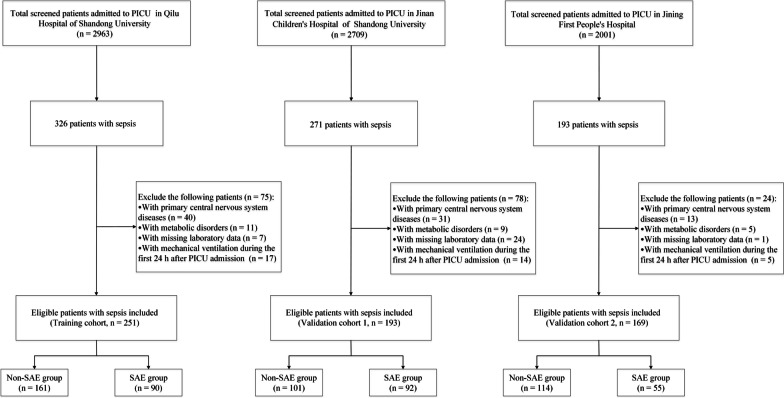
Flowchart of study participants in the training and validation cohorts. SAE: sepsis-associated encephalopathy

The recruitment of the study population is presented in Fig. 1. Among a total of 7673 PICU patients, 790 patients met the diagnosis criteria of sepsis 3.0. After excluding 177 patients, 613 patients with sepsis from three centers in Jinan and Jining were included in the final study. Of these, 251 patients from Qilu Hospital of Shandong University were included as the training cohort, while 193 patients from Jinan Children’s Hospital of Shandong University and 169 patients from Jining First People's Hospital were included as independent external validation cohorts. The patient characteristics of the training and validation cohorts are shown in Additional file 2: Table S2. There were no significant differences in age or sex between the training and validation cohorts (*P* > 0.05). The data on when SAE was diagnosed are shown in Additional file 3: Table S3. Most of the SAE patients were diagnosed within 72 h after PICU admission, while others were diagnosed longer after PICU admission. The earliest time for SAE diagnosis was 8 h, and the average time were 41.0 (interquartile range [IQR] 26.0–61.5) hours, 58.0 (IQR 31.5–91.0) hours, and 48.0 (IQR 25.0–105.0) hours for the training and validation cohorts, respectively. Of the 251 patients with sepsis in the training cohort, 90 (35.9%) were diagnosed with SAE. The baseline and outcome characteristics of the training cohort are presented in Table 1. No significant differences in age or sex were identified between the SAE and non-SAE groups (*P* = 0.773 and *P* = 0.372, respectively). The average age of the patients with SAE was 46.5 (IQR 8.2–99) months, and 53 patients (58.9%) were males. Heart rate (HRs) and respiratory rates (RRs) on PICU admission were significantly higher in the SAE group than in the non-SAE group (HR: 126.5, IQR 109.2–149 vs. 115, IQR 102–131, *P* = 0.004; RR: 30, IQR 25–40 vs. 25, IQR 22–30, *P* < 0.001). However, there were no significant differences between the two groups in other vital signs including systolic pressure, diastolic pressure, and temperature > 38 ℃. No significant differences were observed in suspected infection focus and blood culture positivity between the groups (*P* > 0.05), and both patients with and without SAE displayed a high incidence of respiratory infection (51.1% and 46%, respectively). The main pathogens detected in this population were Staphylococcus, Enterococcus, Streptococcus, *Escherichia coli*, Acinetobacter, Pseudomonas, Klebsiella, and fungi. Patients with SAE seemed to have a higher rate of Gram-negative infections than those without (16.7% vs. 8.7%); however, the difference was not statistically significant (*P* = 0.091). Patients with SAE were more likely to use vasopressors during the PICU stay (36.7% vs. 1.9%, *P* < 0.001). Moreover, patients in the SAE group had a higher incidence of mechanical ventilation during the PICU stay than those in the non-SAE group (33.3% vs. 3.1%). As shown in Table 1, patients with SAE were more likely to suffer from multi-organ dysfunction (87.8% vs. 29.8%, *P* < 0.001), and thus had a higher in-hospital mortality rate (35.6% vs. 0, *P* < 0.001). There were no significant differences in the lengths of hospital and PICU stay (*P* = 0.717 and *P* = 0.892, respectively).

**Table 1:** Baseline and clinical characteristics of the training set

Characteristics	Total (*n* = 251)	Non-SAE group (*n* = 161)	SAE group (*n* = 90)	*P* value
Age (month)	48 (15.5, 95)	48 (18, 94)	46.5 (8.2, 99)	0.773
Gender (male, %)	137 (54.6)	84 (52.2)	53 (58.9)	0.372
Vital signs				
HR (beats/min)	120 (102.5, 139.5)	115 (102, 131)	126.5 (109.2, 149)	0.004
RR (breaths/min)	26 (23, 32)	25 (22, 30)	30 (25, 40)	< 0.001
SBP (mmHg)	99 (85.5, 115)	97 (85, 112)	103.5 (86, 115.8)	0.281
DBP (mmHg)	57 (49, 68)	57 (50, 67)	60 (47.2, 69.8)	0.704
Temperature > 38 ℃, *n* (%)	92 (36.7)	58 (36)	34 (37.8)	0.889
Suspected infection focus, *n* (%)				0.425
Respiratory infection	120 (47.8)	74 (46)	46 (51.1)	
Blood infection	37 (14.7)	23 (14.3)	14 (15.6)	
Urinary tract infection	4 (1.6)	2 (1.2)	2 (2.2)	
Intra-abdominal infection	18 (7.2)	15 (9.3)	3 (3.3)	
Other or unknown	72 (28.7)	47 (29.2)	25 (27.8)	
Blood culture-positive, *n* (%)	40 (15.9)	22 (13.7)	18 (20)	0.256
Microorganisms, *n* (%)	83 (33.1)	48 (29.8)	35 (38.9)	0.185
Gram-positive	35 (13.9)	25 (15.5)	10 (11.1)	0.436
Gram-negative	29 (11.6)	14 (8.7)	15 (16.7)	0.091
Fungus	8 (3.2)	4 (2.5)	4 (4.4)	0.463
Multiple infection (two or more)	18 (7.2)	9 (5.6)	9 (10)	0.297
Vasopressor use during PICU stay time, *n* (%)	36 (14.3)	3 (1.9)	33 (36.7)	< 0.001
Advanced life support during PICU stay time				
Mechanical ventilation, *n* (%)	35 (13.9)	5 (3.1)	30 (33.3)	< 0.001
Renal replacement therapy, *n* (%)	26 (10.4)	13 (8.1)	13 (14.4)	0.170
Outcome				
Multi-organ dysfunction (two or more), *n* (%)	127 (50.6)	48 (29.8)	79 (87.8)	< 0.001
Septic shock, *n* (%)	18 (7.2)	9 (5.6)	9 (10)	0.297
Hospital stay time (days)	12 (7, 21.5)	12 (7, 21)	12 (8, 23)	0.717
PICU stay time (days)	9 (4, 15)	9 (5, 15)	9 (3, 17)	0.892
In-hospital mortality, *n* (%)	32 (12.7)	0 (0)	32 (35.6)	< 0.001

SAE, sepsis-associated encephalopathy; HR, heart rate; RR, respiratory rate; SBP, systolic pressure; DBP, diastolic pressure; PICU, pediatric intensive care unit

### Laboratory information of patients on PICU admission in training cohort

As shown in Table 2, alanine transaminase (ALT), total bilirubin (TBIL), creatine kinase-MB (CK-MB), serum creatinine (Scr), blood urea nitrogen (BUN), lactic dehydrogenase (LDH), prothrombin time (PT), activated partial thromboplastin time (APTT), lactate, arterial partial pressure of carbon dioxide (PaCO_2_), and blood glucose levels were significantly higher in the SAE group than in the non-SAE group (*P* < 0.05). Moreover, lower platelet (PLT) count, albumin, fibrinogen level, and arterial oxygen partial pressure (PaO_2_) were observed in the SAE group (*P* < 0.05). The PCIS was assessed upon PICU admission to evaluate the severity of illness in patients with sepsis. Patients in the SAE group had lower PCIS scores than those in the non-SAE group (90, IQR 80.5–96 vs. 94, IQR 90–96, *P* < 0.001).

**Table 2:** Laboratory information of the training set on PICU admission

Characteristics	Total (*n* = 251)	Non-SAE group (*n* = 161)	SAE group (*n* = 90)	*P* value
WBC (× 10^9^/L)	11.1 (6.1, 20.3)	11.2 (6.4, 20.2)	10.5 (5.5, 21.2)	0.738
PLT (× 10^9^/L)	273 (127, 414)	300 (157, 430)	220.5 (74, 397.8)	0.017
HB (g/L)	103.7 ± 26.1	106.1 ± 23.9	99.3 ± 29.2	0.059
ALT (U/L)	20 (11, 46)	18 (10, 44)	24.5 (13, 59.2)	0.015
TBIL (umol/L)	6.7 (4.3, 11.4)	6.1 (3.8, 10.3)	8.1 (5.1, 16.3)	0.002
Albumin (g/L)	36.9 (31.8, 40.9)	37.7 (33.8, 40.9)	35 (29.1, 41)	0.018
CK-MB (mg/L)	1.5 (0.8, 3.2)	1.2 (0.6, 2.1)	2.5 (1.5, 7.3)	< 0.001
Scr (umol/L)	30 (23, 40.5)	28 (23, 38)	33 (23.2, 53.8)	0.006
BUN (mmol/L)	3.4 (2.5, 4.6)	3.2 (2.4, 3.9)	4 (2.9, 6.1)	< 0.001
LDH (U/L)	384 (270.5, 659)	332 (251, 547)	459.5 (338.8, 1050.2)	< 0.001
Na (mmol/L)	137 (134, 139)	137 (134, 139)	137 (132.2, 139)	0.494
K (mmol/L)	4.2 ± 0.6	4.2 ± 0.6	4.3 ± 0.7	0.263
Fib (g/L)	3 (2.3, 4.1)	3.1 (2.5, 4.1)	2.7 (1.6, 4.1)	0.007
INR	1.2 (1.1, 1.3)	1.2 (1.1, 1.3)	1.2 (1.1, 1.3)	0.157
PT (s)	14 (12.9, 15.6)	13.9 (12.8, 14.9)	14.4 (13, 16.7)	0.006
APTT (s)	36.6 (33.3, 39.7)	35.6 (32, 38.1)	38.8 (35.6, 44)	< 0.001
D-dimer (ug/mL)	1.6 (0.7, 4.8)	1.5 (0.7, 3.8)	1.7 (0.9, 12.4)	0.163
BNP (ng/L)	256 (120, 545)	245 (120, 480)	292.2 (120, 787.2)	0.252
CRP (mg/L)	33.1 (7, 96.9)	34.7 (7.4, 88.9)	31.6 (3.4, 106)	0.464
PCT (ug/L)	0.4 (0.2, 1.4)	0.4 (0.2, 1.3)	0.3 (0.1, 1.5)	0.967
Lactate (mmol/L)	1.4 (1, 2.3)	1.4 (1, 2)	1.6 (1.1, 2.6)	0.030
PH	7.4 (7.4, 7.5)	7.4 (7.4, 7.5)	7.4 (7.4, 7.5)	0.348
PaO_2_ (mmHg)	83 (74, 98)	85 (78, 98)	81 (65, 97)	0.022
PaCO_2_ (mmHg)	34 (28, 38)	33 (27, 37)	36 (31.2, 42)	< 0.001
SaO_2_ (%)	97.9 (95.2, 99)	98 (95.3, 99.1)	97.4 (95.4, 98.9)	0.281
Glucose (mmol/L)	5.8 (5.1, 7.2)	5.7 (5, 6.8)	6.3 (5.2, 8.5)	0.040
PCIS	92 (87, 96)	94 (90, 96)	90 (80.5, 96)	< 0.001

WBC: white blood cell; PLT: platelet; HB: hemoglobin; ALT: alanine transaminase; TBIL: total bilirubin; CK-MB: creatine kinase-MB; Scr: serum creatinine; BUN: blood urea nitrogen; LDH: lactic dehydrogenase; Na: serum sodium; K: serum potassium; Fib: fibrinogen; INR: international normalized ratio; PT: prothrombin time; APTT: activated partial thromboplastin time; BNP: brain natriuretic peptide; CRP: C reactive protein; PCT: procalcitonin; PaO_2_: arterial oxygen partial pressure; PaCO_2_: arterial partial pressure of carbon dioxide; SaO_2_: arterial oxygen saturation; PCIS: pediatric critical illness score

### Independent risk factors associated with the occurrence of SAE

Univariate and multivariate logistic regression showed that independent risk factors for SAE were RR (OR 1.048, 95% CI 1.010–1.090, *P* = 0.015), BUN (OR 1.192, 95% CI 1.079–1.330, *P* = 0.001), APTT (OR 1.108, 95% CI 1.052–1.176, *P* < 0.001), PaCO_2_ (OR 1.056, 95% CI 1.024–1.092, *P* = 0.001), and PCIS (OR 0.954, 95% CI 0.912–0.996, *P* = 0.033) (Table 3).

**Table 3:** Univariate and multivariate logistic analysis of the training set

	Univariate			Multivariate		
	OR	95% CI	*P*	OR	95% CI	*P*
Age (month)	1.001	0.996–1.006	0.687			
Gender (male, %)	1.313	0.779–2.212	0.306			
HR (beats/min)	1.015	1.005–1.026	0.004			
RR (breaths/min)	1.071	1.038–1.105	< 0.001	1.048	1.010–1.090	0.015
SBP (mmHg)	1.005	0.993–1.017	0.436			
DBP (mmHg)	0.994	0.976–1.011	0.473			
Temperature > 38 ℃	1.078	0.632–1.839	0.782			
WBC (× 10^9^/L)	1.004	0.998–1.01	0.157			
PLT (× 10^9^/L)	0.999	0.997–1.000	0.03			
HB (g/L)	0.990	0.980–1.000	0.047			
ALT (U/L)	1.002	1.000–1.003	0.028			
TBIL (µmol/L)	1.011	0.998–1.023	0.097			
Albumin (g/L)	0.948	0.912–0.985	0.007			
CK-MB (mg/L)	1.023	0.998–1.049	0.075			
Scr (µmol/L)	1.002	0.999–1.006	0.186			
BUN (mmol/L)	1.206	1.089–1.336	< 0.001	1.192	1.079–1.330	0.001
LDH (U/L)	1.000	1.000–1.001	0.008			
Na (mmol/L)	0.970	0.923–1.018	0.215			
K (mmol/L)	1.286	0.85–1.946	0.233			
Fib (g/L)	0.790	0.656–0.95	0.013			
INR	2.053	0.866–4.864	0.102			
PT (s)	1.157	1.056–1.267	0.002			
APTT (s)	1.131	1.076–1.188	< 0.001	1.108	1.052–1.176	< 0.001
D-dimer (ug/mL)	1.000	0.999–1.000	0.415			
BNP (ng/L)	1.000	1.000–1.000	0.021			
CRP (mg/L)	1.001	0.997–1.005	0.555			
PCT (µg/L)	1.012	0.993–1.032	0.228			
Lactate (mmol/L)	1.123	1.019–1.238	0.019			
PH	0.236	0.012–4.76	0.346			
PaO_2_ (mmHg)	0.997	0.99–1.004	0.463			
PaCO_2_ (mmHg)	1.065	1.035–1.095	< 0.001	1.056	1.024–1.092	0.001
SaO_2_ (%)	0.973	0.945–1.001	0.059			
Glucose (mmol/L)	1.086	0.993–1.187	0.072			
PCIS	0.913	0.88–0.947	< 0.001	0.954	0.912–0.996	0.033

OR: odds ratio; CI: confidence interval; HR: heart rate; RR: respiratory rate; SBP: systolic pressure; DBP: diastolic pressure; WBC: white blood cell; PLT: platelet; HB: hemoglobin; ALT: alanine transaminase; TBIL: total bilirubin; CK-MB: creatine kinase-MB; Scr: serum creatinine; BUN: blood urea nitrogen; LDH: lactic dehydrogenase; Na: serum sodium; K: serum potassium; Fib: fibrinogen; INR: international normalized ratio; PT: prothrombin time; APTT: activated partial thromboplastin time; BNP: brain natriuretic peptide; CRP: C reactive protein; PCT: procalcitonin; PaO_2_: arterial oxygen partial pressure; PaCO_2_: arterial partial pressure of carbon dioxide; SaO_2_: arterial oxygen saturation; PCIS: pediatric critical illness score

### Development of a prediction nomogram

**Fig. 2:**
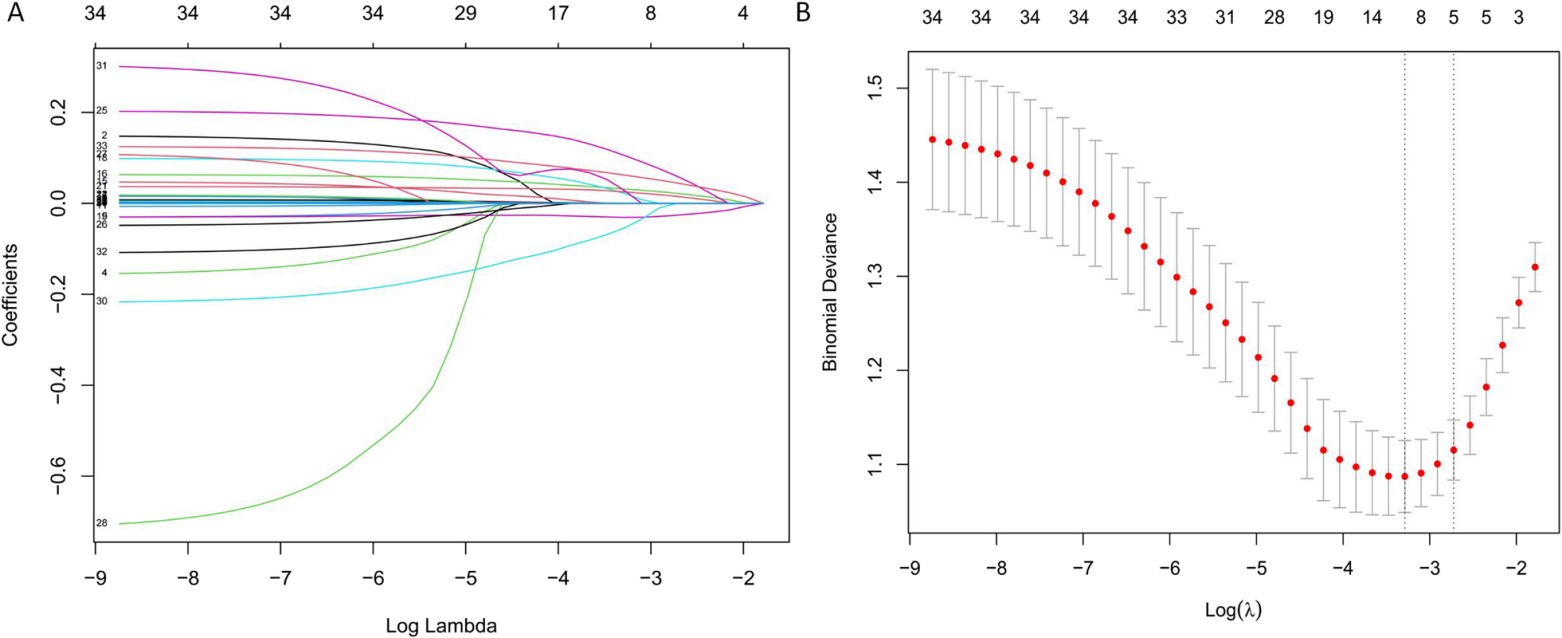
Feature selection using the least absolute shrinkage and selection operator (LASSO) regression in the training cohort. **A** LASSO coefficient profiles of the 34 features. **B** Identification of the optimal penalization coefficient (λ) in the LASSO model was performed via tenfold cross-validation

**Fig. 3:**
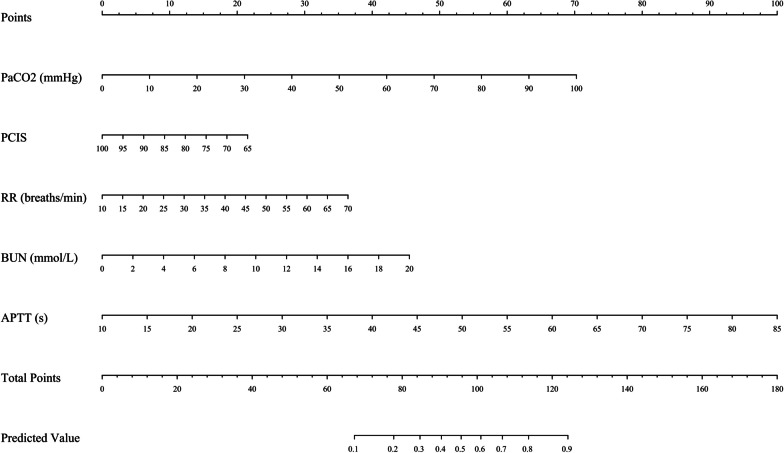
Construction of SAE prediction nomogram in the training cohort. The points of all features were added to obtain the total points, and a vertical line was drawn on the total point to obtain the corresponding ‘predicted value’, which indicates the risk of SAE. PaCO_2_: arterial partial pressure of carbon dioxide; PCIS: pediatric critical illness score; RR: respiratory rate; BUN: blood urea nitrogen; APTT: activated partial thromboplastin time.

Based on the training cohort, a LASSO regression model was established to screen variables for the SAE nomogram (Fig. 2). RR, BUN, APTT, PaCO_2_, and PCIS were identified as predictors in the nomogram. These factors can be used to predict the probability of developing SAE in patients with sepsis, and were presented as a visualization nomogram (Fig. 3). The model established a scoring criteria and assigned a score to each level of prognostication. By summing the scores from each variable, projecting the total scores onto the scale, and drawing a straight line to the bottom of the scale, the nomogram allows users to easily estimate the probability of SAE according to the individual patient characteristics.

### Validation of the nomogram

**Fig. 4:**
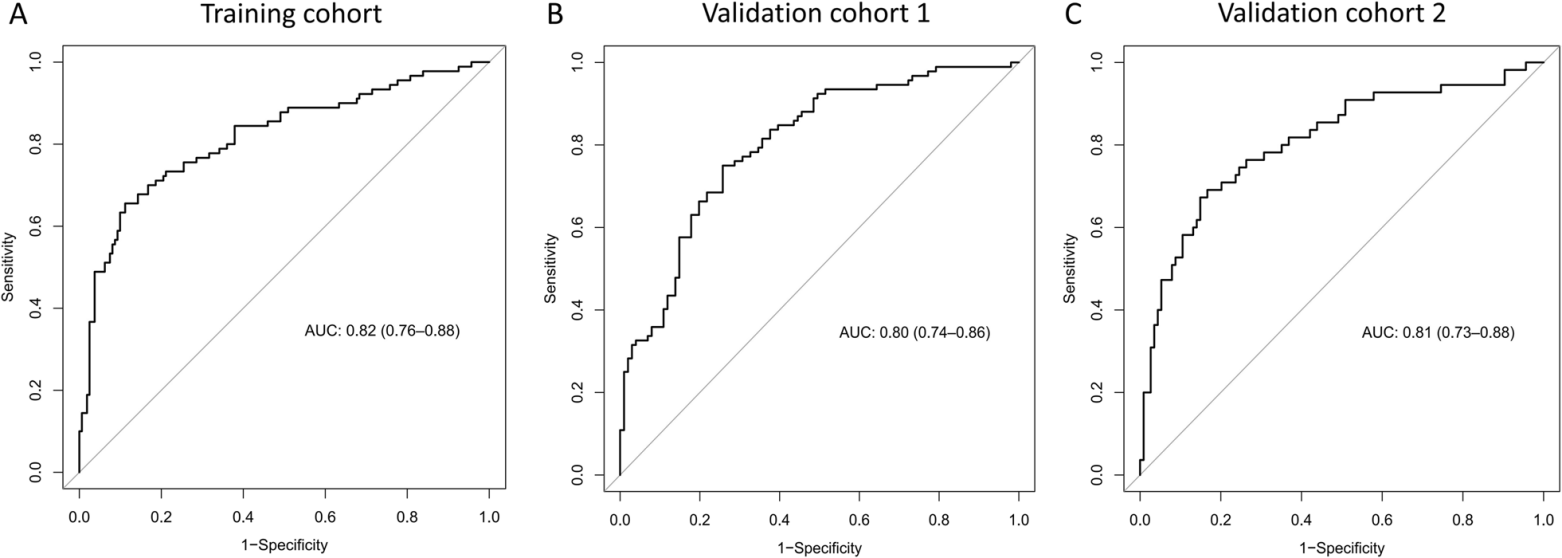
The receiver operating characteristic (ROC) curves of the nomogram in the training cohort (**A**), validation cohort 1 (**B**), and validation cohort 2 (**C**)

**Fig. 5:**
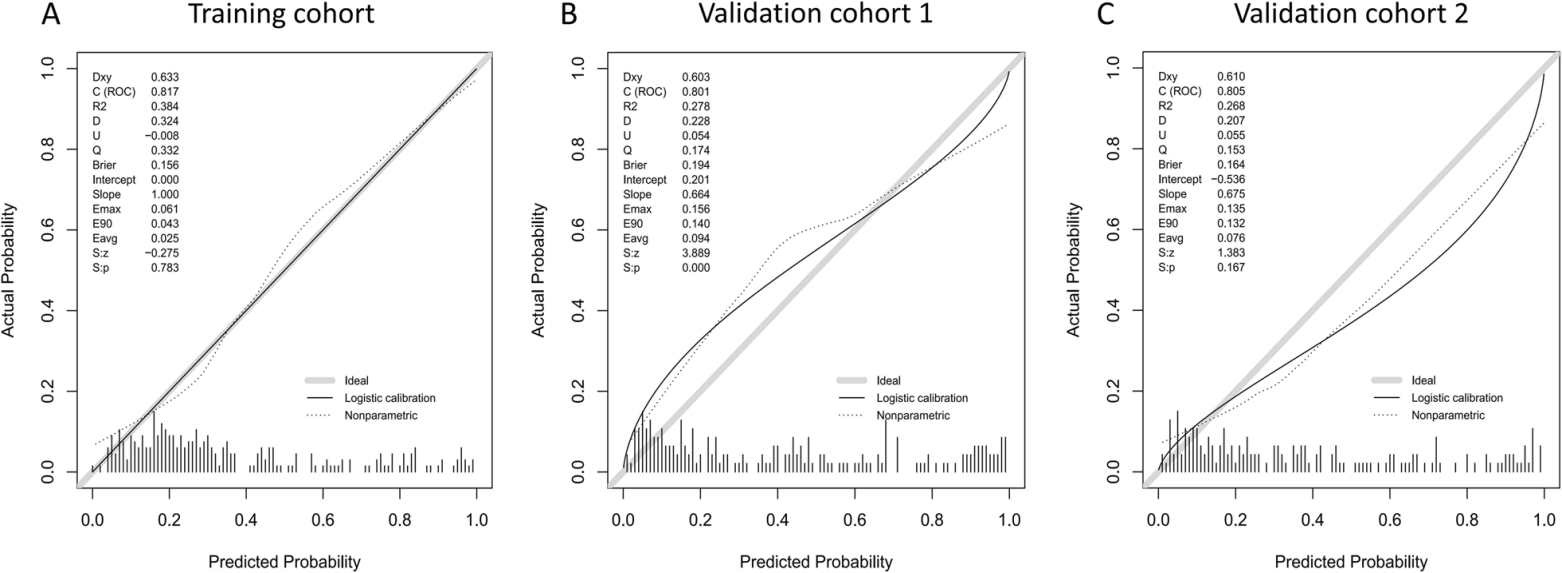
The calibration curves of the nomogram in the training cohort (**A**), validation cohort 1 (**B**), and validation cohort 2 (**C**)

Our model yielded an AUC value of 0.82 (95% CI 0.76–0.88) in the training cohort, with a sensitivity and specificity of 65.6% and 88.8%, respectively (Fig. 4A). Moreover, the calibration plot for the nomogram showed excellent agreement in the SAE probabilities between the observed and predicted values, as both the logistic and nonparametric calibration curves deviated slightly from the ideal line (Fig. 5A). Consistent with the training cohort, in validation cohort 1, the AUC was 0.80 (95% CI 0.74–0.86) for patients, with a sensitivity of 75.0% and a specificity of 74.3% (Fig. 4B). In validation cohort 2, the AUC was 0.81 (95% CI 0.73–0.88) for patients, with a sensitivity of 69.1% and specificity of 83.3% (Fig. 4C). The calibration curves also showed good agreement between the prediction and observation of the risk of SAE in the two external validation cohorts (Fig. 5B, C).

### Clinical use of the nomogram

**Fig. 6:**
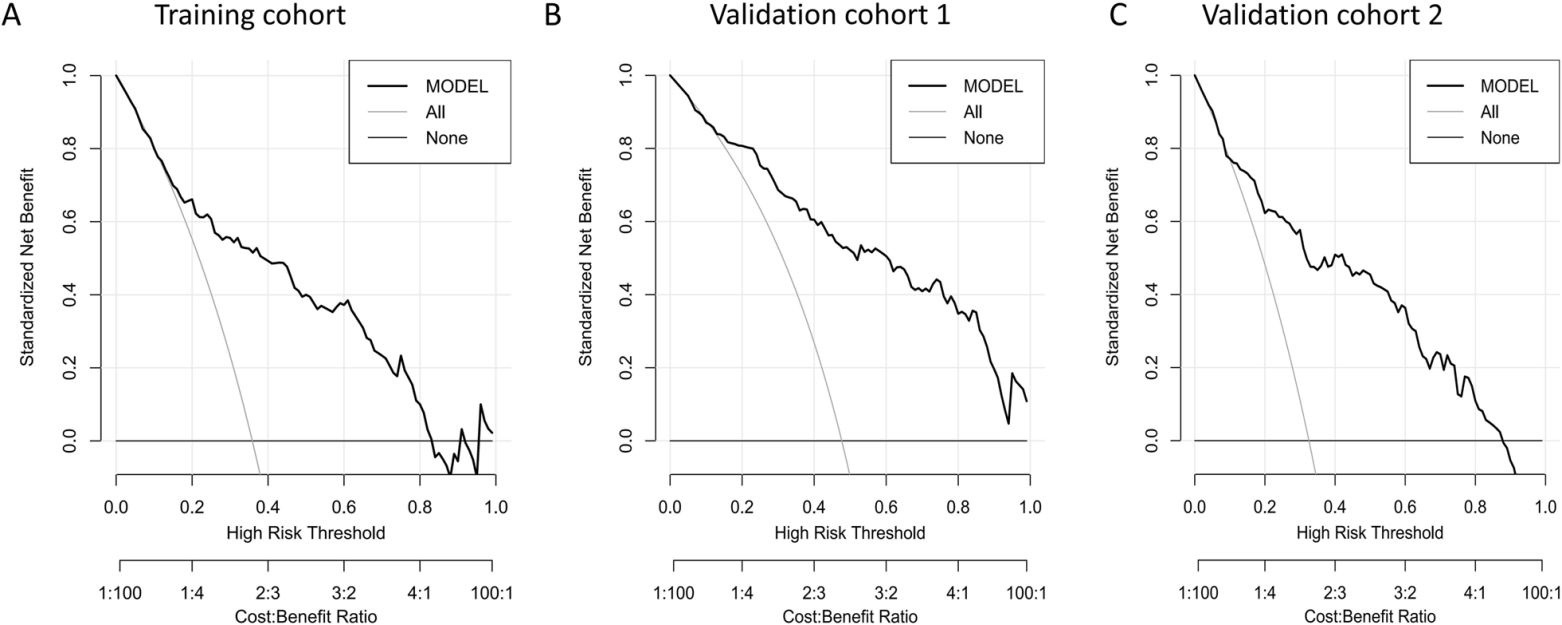
Decision curve analysis (DCA) of the nomogram of SAE. DCA compares the net benefits of three scenarios in predicting the risk of SAE: A perfect prediction model (grey line), screen none (horizontal solid black line), and screen based on the nomogram (ride line). The DCA curves were depicted in the training cohort (**A**), validation cohort 1 (**B**), and validation cohort 2 (**C**)

**Fig. 7:**
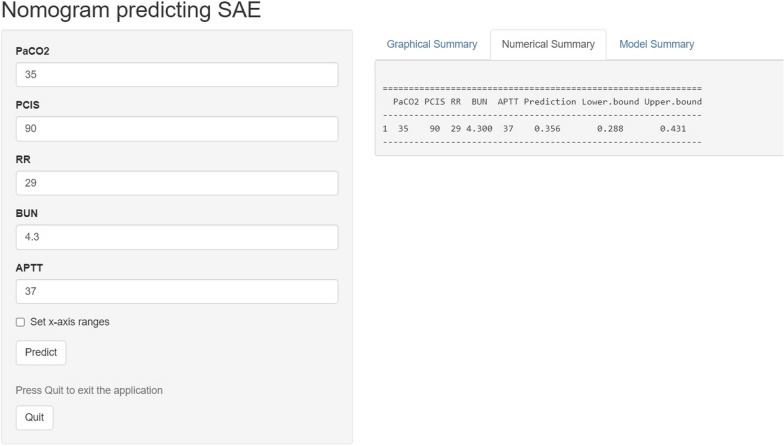
An Internet browser-based online calculator for the SAE prediction nomogram (website: https://johny2020.shinyapps.io/Dyn_SAE/)

The DCA curves for the prediction nomogram in the training and validation cohorts are shown in Fig. 6. According to the DCA, the SAE model of the net benefit had a wide range of threshold probabilities in the training and validation cohorts. To facilitate clinical use, an Excel-based tool or web calculator (https://johny2020.shinyapps.io/Dyn_SAE/) was also developed for the convenience of clinicians (Fig. 7).

## Discussion

Our study evaluated several variables in 613 patients with sepsis in the PICU from multiple centers. RR, BUN, APTT, PaCO_2_, and PCIS were included as predictors of SAE in the nomogram established using a LASSO regression model. The nomogram shows early predictive performance compared with the actual diagnostic time for SAE. A previous study among adult patients with sepsis found that acute renal failure, hypoglycemia, hypercapnia, hypernatremia, and *S. aureus* infection were independent predictors of SAE [19], and the different results from our study demonstrated a difference between adult and pediatric patients. Another retrospective single-center study of 210 pediatric patients with sepsis showed that procalcitonin, Ca^2+^, septic shock, pediatric logistic organ dysfunction score 2 (PELOD-2), and midazolam were independent risk factors for SAE; however, no comprehensive predictive model has been developed [20]. Moreover, our study showed that SAE was associated with higher in-hospital mortality and higher use of ICU resources, confirming the early predictive value of SAE at PICU admission.

PaCO_2_, a vital predictive variable of SAE, was incorporated into the prediction model in our study. Similar results were also reported in another retrospective multicenter analysis, which showed that hypercapnia > 45 mmHg was an independent risk factor for SAE in patients with sepsis upon ICU admission [19]. Carbon dioxide is known to affect cerebral autoregulation in patients with sepsis or septic shock, which may explain why higher PaCO_2_ levels induce SAE [21, 22]. In fact, impaired autoregulation induces increased blood flow in the brain by reducing capillary resistance in the early stage of sepsis, and in the later stages, cerebral hypoperfusion predominates and appears to be a cause of SAE [17]. However, Thees et al. [23] found that carbon dioxide reactivity was not impaired in patients with sepsis syndrome. Therefore, further clinical studies and animal experiments are required to identify the mechanism of carbon dioxide in the occurrence and development of SAE.

As one of the most commonly used indicators of the endogenous coagulation system in clinical practice, APTT was previously shown to be significantly increased in children with SAE compared to those without [20]. Similarly, in the present study, APTT was significantly associated with the occurrence of SAE (*P* < 0.001) and was included in the prediction nomogram because of its strong influence. Several studies have found that increased APTT is a risk factor for death in critically ill children and adults with sepsis [24, 25]. Currently, coagulation disorders are widely considered important causes of microcirculation disturbances, organ dysfunction, and multiple organ failure in sepsis. Overall, APTT was identified as a predictive factor for SAE in our study, and normal coagulation function may be critical in improving the outcomes of patients with SAE.

BUN is a representative indicator of renal function, reflecting the degree of kidney injury and accumulation of metabolic waste, including neurotoxic substances such as antibiotics and hypnotics [19]. Several studies have shown that the accumulation of neurotoxic substances caused by renal insufficiency is one of the risk factors for SAE [7, 19]. Acute renal failure is associated with specific biological alterations, such as severe acidosis and uremia, which may influence brain function [19]. In the present study, we found that BUN had a high predictive value for SAE and was thus used in the prediction model. Another study which included 9126 patients with sepsis reported that BUN was an independent and easily available predictor of 28-day mortality in critically ill patients with sepsis admitted to the ICU [26]. Furthermore, BUN was found to be correlated with the severity and mortality of SAE [20, 27, 28] and proven to be a risk factor related to the in-hospital mortality of patients with sepsis in other models [25, 29]. Accordingly, it is advisable that normal renal function is maintained to reduce the occurrence of SAE and mortality in patients with SAE.

The respiratory rate was also closely associated with SAE in our study. Tachypnea is a compensatory mechanism for tissue hypoxia and metabolic acidosis and reflects the severity of metabolic disorders and tissue hypoxia to a certain extent. As is well known, metabolic disorders, hypoxia and inflammatory cytokine storm are involved in the process of cell damage and permeability increase of endothelial cells, which may be responsible for the occurrence of SAE [17, 30]. Respiratory rate ≥ 22 per minute in sepsis patients can be rapidly identified as an indicator for poor outcome, which was a main part of quick sequential organ failure assessment score (qSOFA) [31]. Respiratory rate has also been included in a model predicting 28-day mortality in patients with severe sepsis and septic shock in the emergency department [32]. Recently, some studies have found that the respiratory rate also shows good predictive value for mortality in patients with SAE [8, 28]. The conclusions of the previous studies further support our results. Therefore, more attention should be paid to patients with sepsis with tachypnea, and measures should be promptly taken to redress their hypoxia and metabolic acidosis, thus reducing the incidence and mortality of SAE.

The PCIS is a quick scoring tool with simple calculations and indices that is widely used for critically ill children in China [33]. Previous studies have shown that PCIS is a valuable predictor of early diagnosis and outcomes in pediatric patients with sepsis [34]. A study of 193 hospitalized children with severe sepsis found that the PCIS and pSOFA had similar predictive values in evaluating patient prognosis [35]. Consistent with previous studies, the present study found that PCIS was a vital risk factor for SAE development in children with sepsis. Moreover, the PCIS showed good accuracy and discriminatory ability in the prognostic prediction of many other critical diseases, such as pneumonia-related bacteremia, hand-foot-and-mouth disease, acute paraquat poisoning, and acute leukemia [36–39]. However, data on the correlation between PCIS and SAE are limited, and future high-quality studies are needed for further exploration.

Other variables were significantly different between the SAE and non-SAE groups, including lactate levels. Elevated serum lactate levels indicate an oxygen supply/demand mismatch and microcirculatory impairment, inducing tissue ischemia and hypoxia in sepsis [40]. Previous studies have suggested that lactate is an important indicator for predicting the prognosis of patients with sepsis and is often used to evaluate disease severity and guide treatment [41]. In patients with septic shock, monitoring serum lactate levels can guide fluid resuscitation and reduce mortality [42]. Many studies have also reported that high lactate levels are significantly associated with mortality in patients with SAE [6, 43]. Moreover, cerebrospinal fluid (CSF) lactate levels in patients with SAE were also increased (2.8–7.9 mmol/L) [17]. In general, the serum lactate level is an important indicator for evaluating the morbidity and prognosis of sepsis and SAE. For patients with lactate acidosis, physicians should provide timely rehydration and other effective treatments to improve tissue perfusion and hypoxia and reduce the morbidity and mortality of SAE.

In the present study, patients with SAE were more likely to use vasopressors during their PICU stay. Vasopressor use indicates the critical state of patients with circulatory dysfunction and hypotension. However, the use of vasopressors, especially for more than 6 h aiming to a relatively higher blood pressure, has been proven to be closely associated with poor prognosis in patients with sepsis [44]. A study related to transcranial Doppler in healthy volunteers showed that norepinephrine, despite increasing arterial pressure, did not increase cerebral perfusion pressure [45]. Another study in healthy subjects suggested that a high dose of norepinephrine may have a negative effect on cerebral oxygenation [46]. Therefore, individualized vasopressor therapy with an appropriate dosage should be applied to patients with SAE.

In our study, respiratory and blood infections were common in children with SAE. However, the primary sites of infection in adult patients with SAE were the respiratory and gastrointestinal tracts [8, 47]. Moreover, Gram-positive bacteria accounted for the largest proportion of adult patients with SAE [8], while *Acinetobacter baumannii* and fungi were more likely found in elderly patients with SAE [48]. However, we found that the most common organisms in children with SAE were Gram-negative bacteria, which was different from that in adult patients. Therefore, clinicians should consider these differences among different age groups when administering antibiotics to patients with SAEs.

This study has some limitations. First, although this was a multicenter study, the vast majority of patients were from Shandong Province, and the sample size was not particularly large. Further studies involving different populations and larger cohorts are required to validate our findings. Second, the retrospective nature of this observational study suggests that unidentified confounding factors may have influenced the results. For example, the vital signs and laboratory data were greatly affected by age, which was not included in the nomogram model. However, we did not build the specific prediction model for each age group because of the small sample size. More patients will be included in our study in order to establish more accurate models for different age groups in the future. Third, a cytokine storm in the central nervous system with an impaired BBB is considered an important pathophysiological mechanism in the development of SAE [17]. Unfortunately, data regarding CSF cytokine were available only in a few of the studied patients due to the fact that this test was not routinely conducted in many hospitals. We built the nomogram model using predictive factors that are easier to obtain.

## Conclusion

In summary, a prediction nomogram for SAE based on PaCO_2_, APTT, BUN, RR, and PCIS was developed and validated externally. The nomogram and the corresponding online calculator can be conveniently used to accurately predict the morbidity of SAE. This may be beneficial for the early recognition and management of SAE, and ultimately improve the prognosis of patients with SAE.

### Supplementary Information

**Additional file 1: Table S1.** TRIPOD Checklist: prediction model development.

**Additional file 2: Table S2.** Baseline and clinical characteristics of the study cohorts.

**Additional file 3: Table S3.** Grouping of the patients with SAE by the timing of SAE diagnosis after PICU admission.

## Data Availability

The datasets used or analyzed in the current study are available from the corresponding author upon reasonable request**.**
