# Supplementary material for: Development and validation of a nomogram to predict the risk of sepsis-associated encephalopathy for septic patients in PICU: a multicenter retrospective cohort study
Source: J Intensive Care. 2024 Feb 20;12:8. doi: 10.1186/s40560-024-00721-7 (PMC10877756; doi:10.1186/s40560-024-00721-7)
Supplement: Supplementary file 2 — Additional file 2: Table S2. Baseline and clinical characteristics of the study cohorts. [file 40560_2024_721_MOESM2_ESM.pdf]

Supplementary Table 2 Baseline and clinical characteristics of the study cohorts.

Characteristics	Training cohort （n = 251）	Validation cohort 1 （n = 193）	Validation cohort 2 （n = 169）	*P* value
Age (month)	48 (15.5, 95)	45 (13, 88)	42 (9, 96)	0.258
Gender (male, %)	137 (54.6)	107 (55.4)	101 (59.8)	0.554
Vital signs				
HR (beats/min)	120 (102.5, 139.5)	132 (95, 153)	124 (99, 144)	0.176
RR (breaths/min)	26 (23, 32)	27 (22, 34)	28 (21, 33)	0.755
SBP (mmHg)	99 (85.5, 115)	97 (84, 110)	95 (85, 115)	0.359
DBP (mmHg)	57 (49, 68)	53 (45, 67)	59 (45, 69)	0.123
Temperature > 38℃, n (%)	92 (36.7)	81 (42)	75 (44.4)	0.25
WBC ($\times 10^9$ /L)	11.1 (6.1, 20.3)	11.4 (6, 19.3)	11.6 (6.7, 19.3)	0.851
PLT ($\times 10^9$ /L)	273 (127, 414)	230 (132, 341)	230 (128, 352)	0.298
HB (g/L)	103.7 ± 26.1	101 ± 23.9	106.2 ± 22.7	0.136
ALT (U/L)	20 (11, 46)	20 (13, 55)	23 (13.1, 56.7)	0.229
TBIL (umol/L)	6.7 (4.3, 11.4)	7 (4.7, 14.9)	7.8 (4.8, 12.7)	0.348
Albumin (g/L)	36.9 (31.8, 40.9)	35.8 (30.6, 40.1)	37.1 (33.7, 41)	0.089
CK-MB (mg/L)	1.5 (0.8, 3.2)	1.6 (0.7, 8.9)	1.5 (0.8, 11)	0.209
Scr (umol/L)	30 (23, 40.5)	32.4 (20.9, 55)	33 (23, 60.2)	0.111
BUN (mmol/L)	3.4 (2.5, 4.6)	3.3 (2.3, 5.1)	3.2 (2.1, 5.1)	0.65
LDH (U/L)	384 (270.5, 659)	443 (261, 747)	396 (270, 755)	0.883
Na (mmol/L)	137 (134, 139)	136 (133, 140)	137 (133, 140)	0.879

K (mmol/L)	4.2 (3.8, 4.6)	4.1 (3.7, 4.7)	4.2 (3.6, 4.8)	0.706
Fib (g/L)	3 (2.3, 4.1)	2.8 (1.9, 4)	2.7 (1.7, 4)	0.077
INR	1.2 (1.1, 1.3)	1.2 (1, 2)	1.2 (1, 1.7)	0.074
PT (s)	14 (12.9, 15.6)	14 (12, 19)	15 (12, 17)	0.768
APTT (s)	36.6 (33.3, 39.7)	36 (29, 44.5)	36 (31, 45)	0.858
D-dimer (ug/mL)	1.6 (0.7, 4.8)	1.9 (0.8, 3.5)	2 (0.7, 5.3)	0.399
BNP (ng/L)	256 (120, 545)	292.1 (122, 1088)	236 (112, 974)	0.238
CRP (mg/L)	33.1 (7, 96.9)	48.3 (7.6, 125)	44.5 (6.2, 113.9)	0.246
PCT (ug/L)	0.4 (0.2, 1.4)	0.4 (0.2, 3.1)	0.4 (0.2, 3.2)	0.066
Lactate (mmol/L)	1.4 (1, 2.3)	1.5 (0.7, 2.3)	1.3 (1, 2.9)	0.177
PH	7.4 (7.4, 7.5)	7.4 (7.3, 7.5)	7.4 (7.3, 7.5)	0.081
PaO_2 (mmHg)	83 (74, 98)	89 (68, 100)	85 (68, 127)	0.23
$PaCO_2$ (mmHg)	34 (28, 38)	35 (28, 43)	34 (28, 39)	0.198
SaO_2 (%)	97.9 (95.2, 99)	98 (94, 99)	97.7 (95.3, 99)	0.974
Glucose (mmol/L)	5.8 (5.1, 7.2)	6 (4.7, 7.4)	5.8 (4.6, 7.3)	0.516
PCIS	92 (87, 96)	92 (86, 96)	92 (86, 96)	0.344

HR, heart rate; RR, respiratory rate; SBP, systolic pressure; DBP, diastolic pressure; PICU, pediatric intensive care unit; WBC, white blood cell; PLT, platelet; HB, hemoglobin; ALT, alanine transaminase; TBIL, total bilirubin; CK-MB, creatine kinase-MB; Scr, serum creatinine; BUN, blood urea nitrogen; LDH, lactic dehydrogenase; Na, serum sodium; K, serum potassium; Fib, fibrinogen; INR, international normalized ratio; PT, prothrombin time; APTT, activated partial thromboplastin time; BNP, brain natriuretic peptide; CRP, C reactive protein; PCT, procalcitonin; PaO_2, arterial oxygen partial pressure; $PaCO_2$, arterial partial pressure of carbon dioxide; SaO_2, arterial oxygen saturation; PCIS, pediatric critical illness score.
