# Supplementary material for: Development and validation of a nomogram to predict the risk of sepsis-associated encephalopathy for septic patients in PICU: a multicenter retrospective cohort study
Source: J Intensive Care. 2024 Feb 20;12:8. doi: 10.1186/s40560-024-00721-7 (PMC10877756; doi:10.1186/s40560-024-00721-7)
Supplement: Supplementary file 3 — Additional file 3: Table S3. Grouping of the patients with SAE by the timing of SAE diagnosis after PICU admission. [file 40560_2024_721_MOESM3_ESM.pdf]

Supplementary Table 3 Grouping of the patients with SAE by the timing of SAE diagnosis after PICU admission.

The timing of SAE diagnosis after PICU admission	Training cohort （n = 90）	Validation cohort 1 （n = 92）	Validation cohort 2 （n = 55）
< 24 h	18 (20.0)	15 (16.3)	13 (23.6)
24 ≤ x< 72 h	54 (60.0)	40 (43.5)	22 (40.0)
72 ≤ x< 168 h	15 (16.7)	27 (29.3)	12 (21.8)
≥ 168 h	3 (3.3)	10 (10.9)	8 (14.5)

SAE, sepsis-associated encephalopathy; PICU, pediatric intensive care unit.
